# AUTONOMOUS BLADDER TRAINING FOR NEUROGENIC BLADDER: A RANDOMIZED CONTROLLED TRIAL

**DOI:** 10.2340/jrm.v58.45818

**Published:** 2026-07-23

**Authors:** Yue YANG, Lianchi LI, Jing YE, Hongxia PAN, Jianmei ZHANG, Li ZENG, Yuyang LONG, Xinling GAN

**Affiliations:** 1 Rehabilitation Medicine Center and Institute of Rehabilitation Medicine, West China Hospital, Sichuan University, Chengdu; 2 Key Laboratory of Rehabilitation Medicine in Sichuan Province, West China Hospital, Sichuan University, Chengdu; 3 Department of Rehabilitation Medicine, West China Hospital, Sichuan University/West China School of Nursing, Sichuan University, Chengdu; 4 Department of Critical Care Medicine, West China Hospital, Sichuan University, Chengdu, China

**Keywords:** neurogenic bladder, autonomous bladder function training, urodynamic parameters, urinary tract infection, randomized controlled trial

## Abstract

**Background:**

Neurogenic bladder causes impaired bladder emptying, urinary complications, and reduced quality of life. This trial evaluated whether structured multimodal autonomous bladder training improves bladder function in patients with neurogenic bladder.

**Methods:**

In this single-centre randomized controlled trial, 168 adults were assigned 1:1 to autonomous bladder training plus routine rehabilitation care or routine care alone. The 4-week intervention included induced voiding, sensory awareness training, pelvic floor muscle training, bladder–sphincter coordination exercises, and bladder desensitization. The primary outcome was change in post-void residual urine volume (RUV) at 4 weeks. Secondary outcomes included urodynamic parameters, Neurogenic Bladder Symptom Score (NBSS), symptomatic urinary tract infection (UTI) requiring antibiotics, and adverse events.

**Results:**

At 4 weeks, RUV improved more with autonomous bladder training than with routine care (adjusted mean difference in change, –55.8 mL; 95% CI, –64.7 to –46.9). Maximum urinary flow rate, maximum detrusor pressure, and NBSS also favoured the intervention. RUV reduction ≥50 mL occurred in 79.8% versus 42.9% of participants. Symptomatic UTI requiring antibiotics occurred in 9.5% versus 27.4%, although urine culture confirmation was incomplete. Exploratory 12-week follow-up suggested partial maintenance of benefit. Adverse events were uncommon, and no serious adverse events occurred.

**Conclusions:**

Autonomous bladder training improved short-term urodynamic and patient-reported outcomes, with exploratory evidence of partial benefit maintenance at 12 weeks. Infection-related findings require cautious interpretation because culture confirmation was incomplete. Longer multicentre trials are warranted.

## LAY ABSTRACT

People with neurogenic bladder often have difficulty emptying their bladder because of damage to the nervous system. This can lead to repeated urinary problems, discomfort, and a reduced quality of life. We studied whether a structured bladder training programme could help these patients regain better bladder control. In this study, 168 adults received either the training programme together with usual rehabilitation care or usual care alone. After 4 weeks, people who received the training were able to empty their bladder more effectively and reported fewer bladder-related symptoms. They also had fewer urinary infections requiring antibiotic treatment, although this finding needs further confirmation. Some benefits were still present at 12 weeks. These findings suggest that structured bladder training may be a useful addition to routine rehabilitation and may help patients manage their bladder more independently.

Neurogenic bladder (NB) is a complex dysfunction of the urinary bladder caused by neurological impairment, such as spinal cord injury (SCI), multiple sclerosis, or other central nervous system disorders, leading to disruptions in storage and voiding functions (1). It is estimated that up to 80% of patients with SCI develop NB, which often manifests as urinary retention, incontinence, or elevated residual urine volume (2). Without effective management, NB can progress to severe complications, including recurrent urinary tract infections (UTIs), hydronephrosis, vesicoureteral reflux, and eventually renal failure – one of the leading causes of mortality in this population (3, 4).

Current guidelines recommend clean intermittent catheterization (CIC) as the gold standard for bladder management in NB patients. Evidence indicates that CIC not only reduces the incidence of UTIs but also promotes bladder function recovery more effectively than indwelling catheters (5). A recent large-scale multicentre retrospective cohort study demonstrated that patients using CIC were twice as likely to regain voluntary voiding function within 1 year compared with those with indwelling catheters, underscoring the importance of catheterization strategies in functional rehabilitation (6). However, CIC alone often fails to address multifaceted challenges such as impaired detrusor contractility, poor bladder–sphincter coordination, and persistent high post-void residual urine (7, 8).

In recent years, there has been growing emphasis on integrating non-instrumental bladder training techniques into standard NB rehabilitation protocols. These include triggered voiding, pelvic floor muscle training, bladder desensitization, and mindfulness-based voiding exercises, which aim to enhance bladder sensation, promote voluntary control, and facilitate neural reorganization (9–11). Such autonomous bladder function training strategies have shown promise in improving urodynamic parameters and reducing urinary complications, particularly when combined with CIC. However, evidence remains fragmented, and well-designed randomized controlled trials (RCTs) evaluating the efficacy and safety of structured multimodal bladder training programmes in the NB population are lacking.

In China, the adoption of bladder rehabilitation techniques remains suboptimal, with limited integration of systematic training into routine care. Existing interventions often focus narrowly on catheterization or pharmacological management, overlooking potential synergies between mechanical drainage and functional training. This gap is critical, as recent studies emphasize the need for personalized, function-oriented approaches addressing both physiological and psychosocial dimensions of NB (12, 13).

To address these limitations, we developed a novel multimodal intervention – Autonomous Bladder Function Training – which integrates induced voiding, pelvic floor muscle training, bladder–sphincter coordination exercises, and desensitization protocols into a structured, patient-tailored programme. This approach aims to improve bladder emptying, reduce residual urine, and enhance patients’ self-management skills and quality of life. Through a randomized controlled trial, we evaluated the efficacy and safety of this training strategy in patients with NB, with the goal of providing high-level evidence to support its integration into standard neurogenic bladder rehabilitation.

## MATERIALS AND METHODS

### Study design

This prospective, single-centre, randomized controlled trial was conducted in the Rehabilitation Medicine Department of West China Hospital between August 2023 and July 2025. Eligible participants were randomly assigned in a 1:1 ratio to autonomous bladder training plus routine rehabilitation care or routine rehabilitation care alone. The trial was approved by the Biomedical Ethics Review Committee of West China Hospital of Sichuan University and registered in the Chinese Clinical Trial Registry (ChiCTR2500115185). The full study protocol and statistical analysis plan were finalized before data analysis and are available from the corresponding author on reasonable request. A summary of protocol-specified outcomes and the statistical analysis plan is provided in Appendix S1.

### Eligibility criteria

Inclusion criteria were as follows: adult patients aged ≥ 18 years diagnosed with neurogenic bladder according to the Comprehensive Classification Standard for Upper and Lower Urinary Tract Dysfunction in Patients with Neurogenic Bladder; presentation during the subacute or chronic phase of neurological injury; and at least 1 of the following clinical manifestations: post-void residual urine volume > 100 mL, urinary incontinence, or urinary retention. Additional requirements included haemodynamic stability (absence of sustained hypotension or uncontrolled hypertension) and provision of written informed consent by the patient or their legal representative.

Exclusion criteria included: severe comorbidities such as significant acid-base or electrolyte imbalances, or impaired function of major organs (heart, lungs, kidneys); active severe UTI, coagulation disorders, or communicable diseases; history of major urological surgery (e.g., urethral sphincterotomy or cystostomy); bladder safe capacity < 100 mL or presence of bladder perforation; frequent autonomic dysreflexia, sustained bladder pressure > 40 cmH_2_O, or vesicoureteral reflux; impaired consciousness, diagnosed psychiatric disorders, or clinically unstable medical conditions; requirement for large-volume fluid resuscitation; and unwillingness to provide informed consent or comply with study procedures. (Detailed criteria are provided in Table SI.)

### Recruitment and consent

Potential participants were identified and referred to the study team by clinicians from the Rehabilitation Medicine Department. After eligibility screening, written informed consent was obtained from all participants. For patients unable to provide consent due to cognitive or communication impairments, consent was secured from their legally authorized representatives. The study protocol was reviewed and approved by the Ethics Committee of West China Hospital of Sichuan University.

### Randomization and allocation

Eligible participants were randomly assigned in a 1:1 ratio to the autonomous bladder training group or the routine care control group. The allocation sequence was generated by an independent statistician who was not involved in participant recruitment, intervention delivery, outcome assessment, or data analysis, using a computer-generated blocked randomization procedure with randomly varying block sizes of 4 and 6. No stratification was used. The allocation sequence was implemented through a secure web-based allocation system. After written informed consent and baseline assessment were completed, the attending clinician accessed the system to obtain the assignment. Allocation was therefore concealed until the point of assignment. Because of the behavioural nature of the intervention, participants and treating therapists could not be blinded; however, urodynamic assessors, data managers, and statisticians remained blinded to group allocation until the primary analysis was completed.

### Sample size calculation

A target sample size of 150 participants (75 per group) was set based on the anticipated patient availability and feasibility within the study period at our single centre. Based on preliminary data (mean RUV of 110 ± 45 mL in the intervention group vs 155 ± 40 mL in controls, effect size *d* = 1.09), this sample provides > 99% power (α = 0.05, two-sided *t*-test) to detect the expected difference. It also maintains > 90% power for a more conservative effect size (*d* = 0.73).

### Study procedures

The intervention group received a structured, multimodal autonomous bladder training programme daily for 4 weeks. The intervention was designed as an integrated rehabilitation package rather than a single-component therapy because neurogenic bladder dysfunction commonly involves multiple interacting mechanisms, including impaired bladder sensation, reduced voluntary voiding control, detrusor–sphincter dyssynergia, pelvic floor dysfunction, and incomplete bladder emptying. The protocol included: (1) induced voiding using auditory stimulation and thermal stimulation to the suprapubic region; (2) voiding awareness and sensory training using mindfulness-based imagery before catheterization; (3) pelvic floor muscle training using sustained anal sphincter contractions; (4) bladder–sphincter coordination training through therapist-delivered digital anal stretching; and (5) bladder desensitization training via post-void instillation of 100 mL of alternating warmed and room-temperature sterile saline. To improve reproducibility, the intervention was delivered according to a standardized operating manual and the detailed protocol is presented in Table SII. All therapists and rehabilitation nurses received protocol-specific training before study initiation. Treatment sessions were documented using standardized therapy logs, including completion of each component, session duration, patient tolerance, and adverse events.

The control group received standard routine rehabilitation care over the same 4-week period. This included clean intermittent catheterization (CIC) for eligible patients and indwelling catheterization when clinically indicated, both performed according to established hospital guidelines. In addition, patients were provided with structured health education on fluid management, catheter care, and prevention of urinary complications, as well as basic psychological support through regular nursing consultations. Care was delivered by the same clinical team to ensure consistency in routine management across both groups.

Both groups underwent identical evaluation protocols at baseline and at the 4-week follow-up. Assessments included comprehensive urodynamic studies, routine urinalysis, urine culture when clinically indicated, bladder ultrasound, and patient-reported outcome measures using the Neurogenic Bladder Symptom Score (NBSS). In addition, participants were followed for an exploratory post-intervention period up to 12 weeks after randomization. The 12-week follow-up was conducted through outpatient visits or structured telephone interviews when in-person assessment was not feasible. Long-term exploratory outcomes included maintenance of RUV improvement, NBSS, catheterization frequency, clinically diagnosed symptomatic UTI requiring antibiotic treatment, UTI-related hospitalization, and late adverse events. Urodynamic reassessment at 12 weeks was performed when clinically feasible, whereas patient-reported outcomes and infection-related events were collected for all participants through medical records and structured follow-up.

### Data collection and management

The prespecified primary outcome was change in RUV from baseline to 4 weeks. Key secondary urodynamic outcomes included changes in maximum urinary flow rate and maximum detrusor pressure. Other secondary outcomes included clinically diagnosed symptomatic UTI requiring antibiotic treatment, NBSS, patient satisfaction, and adverse events. Responder outcomes were included to aid clinical interpretation: RUV reduction of at least 50 mL, achievement of RUV below 100 mL, NBSS reduction of at least 5 points, reduction in catheterization frequency by at least once per day, and spontaneous voiding at least once per day.

Clinically diagnosed symptomatic UTI requiring antibiotic treatment was defined as new or worsening urinary tract-related symptoms or systemic symptoms judged by the treating physician to be compatible with UTI, together with supportive urinalysis findings and initiation of antibiotic therapy. Urinary tract-related symptoms included new or worsening urgency, frequency, suprapubic discomfort, increased urinary incontinence, cloudy or malodorous urine accompanied by clinical concern, or increased catheterization difficulty. Systemic symptoms included fever, chills, malaise, or unexplained worsening of spasticity or autonomic dysreflexia.

Urinalysis was used as supportive evidence rather than as the sole diagnostic criterion because pyuria and bacteriuria are common in patients with NB managed with CIC. Urine culture was obtained when clinically indicated; however, culture confirmation was not systematically available for all suspected episodes. Therefore, the infection-related outcome was analysed as clinically diagnosed symptomatic UTI requiring antibiotic treatment rather than strictly culture-confirmed UTI. Culture-positive symptomatic UTI was reported as a supportive exploratory analysis. Urine samples were preferably collected before the daily bladder desensitization procedure and before saline instillation to reduce the potential influence of mechanical washout. Samples collected immediately after bladder irrigation or saline instillation were avoided whenever possible; however, exact timing relative to catheterization and saline instillation could not be fully standardized in all cases.

### Safety monitoring and adverse events

Adverse events were collected systematically throughout the 4-week intervention period in both groups using a predefined monitoring form. Trained rehabilitation nurses assessed and recorded adverse events at each daily treatment session and during routine clinical care. Prespecified adverse events included symptomatic UTI requiring antibiotic treatment, autonomic dysreflexia, haematuria, catheter-related trauma, suprapubic pain, haemodynamic instability, and any event requiring interruption or discontinuation of the intervention. Serious adverse events were defined as events resulting in death, life-threatening deterioration, prolonged hospitalization, persistent disability, or other medically important conditions. Severity and intervention-relatedness were assessed by the treating physician and reviewed by the principal investigator.

### Statistical analysis

Analyses were performed according to the intention-to-treat principle using SPSS 22.0 (IBM Corp, Armonk, NY, USA). The primary analysis compared change in RUV from baseline to 4 weeks between groups using analysis of covariance, with treatment group as the main effect and baseline RUV as a covariate. Continuous secondary outcomes were analysed similarly when baseline values were available, and categorical outcomes were summarized as risk ratios or risk differences with 95% confidence intervals. The primary outcome was tested at a two-sided alpha level of 0.05. The Holm–Bonferroni method was applied to key secondary urodynamic outcomes to account for multiple comparisons. Other secondary and exploratory outcomes, including symptomatic UTI, responder outcomes, 12-week outcomes, and patient-reported outcomes, were interpreted cautiously and exploratorily.

Participant retention and outcome-level missingness were assessed separately. Missing data were summarized by variable and treatment group. For continuous outcomes with missing 4-week values, multiple imputation by chained equations was performed under a missing-at-random assumption. The imputation model included treatment group, baseline value of the outcome, age, sex, aetiology of NB, disease phase, baseline RUV, catheterization method, and other available 4-week outcomes. Twenty imputed datasets were generated and pooled using Rubin’s rules. Group allocation was included in the imputation model but was not imputed. Complete-case analyses and a conservative worst-case analysis for the primary outcome were performed as sensitivity analyses.

Because NB may differ according to neurological aetiology, exploratory subgroup analyses compared participants with SCI and those with non-SCI aetiologies. Treatment-by-subgroup interaction terms were tested in the ANCOVA model for the primary outcome. These analyses were exploratory and were not adjusted for multiplicity. Twelve-week outcomes were analysed as exploratory post-intervention outcomes and interpreted descriptively.

## RESULTS

### Participant flow and baseline characteristics

**Fig. 1:**
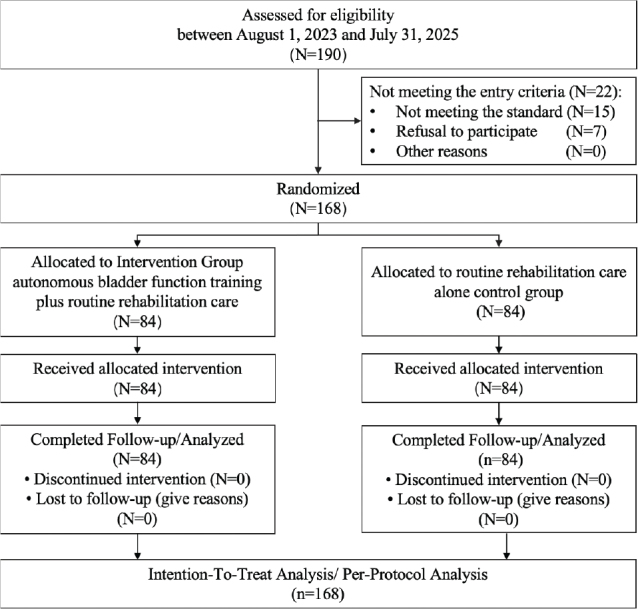
Participant flow diagram.

Between August 2023 and January 2025, 190 patients were assessed for eligibility; 22 were excluded, and 168 were randomized (84 per group) (Fig. 1). All randomized participants completed the 4-week follow-up and were included in the intention-to-treat population. No participant discontinued the assigned intervention or was lost to follow-up. Outcome-level missing data were infrequent and balanced between groups. Missing 4-week values occurred in 2 participants for RUV, 3 for maximum flow rate, 4 for maximum detrusor pressure, 2 for NBSS, and 1 for patient satisfaction. Clinically diagnosed symptomatic UTI and adverse-event status were available for all participants. Results were similar in complete-case and multiple-imputation analyses. Baseline characteristics were well balanced between groups (Table I).

**Table I:** Baseline characteristics of the patients on enrolment

Characteristic	Autonomous bladder training group (*n* = 84)	Routine care control group (*n* = 84)	All patients (*n* = 168)	*p*-value
Demographics				
Age, years, mean ± SD	45.6 ± 12.3	47.1 ± 11.8	46.4 ± 12.0	0.42
Age group, *n* (%)				0.65
< 40 years	25 (29.8%)	22 (26.2%)	47 (28.0%)	–
40–60 years	45 (53.6%)	48 (57.1%)	93 (55.4%)	–
> 60 years	14 (16.7%)	14 (16.7%)	28 (16.7%)	–
Male sex, *n* (%)	52 (61.9%)	49 (58.3%)	101 (60.1%)	0.65
Body mass index (kg/m^2^), mean ± SD	23.8 ± 4.1	24.1 ± 3.8	24.0 ± 3.9	0.6
Aetiology of neurogenic bladder, *n* (%)				0.78
Spinal cord injury (SCI)	65 (77.4%)	63 (75.0%)	128 (76.2%)	–
Multiple sclerosis	10 (11.9%)	12 (14.3%)	22 (13.1%)	–
Stroke	5 (6.0%)	4 (4.8%)	9 (5.4%)	–
Other neurological disorders	4 (4.8%)	5 (6.0%)	9 (5.4%)	–
Time since neurological injury				0.88
Subacute (1–6 months), *n* (%)	48 (57.1%)	46 (54.8%)	94 (56.0%)	–
Chronic (> 6 months), *n* (%)	36 (42.9%)	38 (45.2%)	74 (44.0%)	–
Severity and comorbidity scores				
ASIA impairment scale (for SCI patients only), *n* (%)	(*n* = 65)	(*n* = 63)	(*n* = 128)	0.82
Grade A (complete)	28 (43.1%)	25 (39.7%)	53 (41.4%)	–
Grade B/C/D (incomplete)	37 (56.9%)	38 (60.3%)	75 (58.6%)	–
Charlson comorbidity index, mean ± SD	1.2 ± 1.5	1.4 ± 1.6	1.3 ± 1.5	0.4
Baseline urodynamic parameters				
Post-void residual volume (mL), mean ± SD	158.2 ± 41.5	155.8 ± 39.2	157.0 ± 40.3	0.69
Maximum flow rate (mL/s), mean ± SD	9.5 ± 2.8	9.8 ± 3.0	9.7 ± 2.9	0.5
Maximum detrusor pressure (cmH_2_O), mean ± SD	52.3 ± 10.1	50.9 ± 9.6	51.6 ± 9.8	0.35
Bladder compliance (mL/cmH_2_O), Mean ± SD	28.5 ± 12.1	29.8 ± 11.4	29.2 ± 11.7	0.47
Baseline bladder management, *n* (%)				0.92
Clean intermittent catheterization (CIC)	70 (83.3%)	69 (82.1%)	139 (82.7%)	–
Indwelling catheter	14 (16.7%)	15 (17.9%)	29 (17.3%)	–
Comorbidities, *n* (%)				
Hypertension	18 (21.4%)	16 (19.0%)	34 (20.2%)	0.7
Diabetes mellitus	12 (14.3%)	10 (11.9%)	22 (13.1%)	0.64
Recurrent urinary tract infection	25 (29.8%)	28 (33.3%)	53 (31.5%)	0.62
Concurrent medications, *n* (%)				
Anticholinergics	45 (53.6%)	48 (57.1%)	93 (55.4%)	0.65
Alpha-blockers	20 (23.8%)	18 (21.4%)	38 (22.6%)	0.72
Laboratory values at baseline				
Serum creatinine (μmol/L), mean ± SD	68.5 ± 20.1	71.2 ± 22.5	69.9 ± 21.3	0.41
eGFR (mL/min/1.73m^2^), mean ± SD	90.5 ± 18.8	88.1 ± 17.2	89.3 ± 18.0	0.38

SD: dtandard feviation; SCI: spinal cord injury; ASIA: American Spinal Injury Association; eGFR: estimated Glomerular Filtration Rate.

*p*-values are for comparisons between the Autonomous Bladder Training Group and the Routine Care Control Group; *p*-values for continuous variables were derived from independent samples *t*-tests; *p*-values for categorical variables were derived from χ^2^ tests (or Fisher’s exact test when appropriate).

### Primary and key secondary urodynamic outcomes

**Fig. 2:**
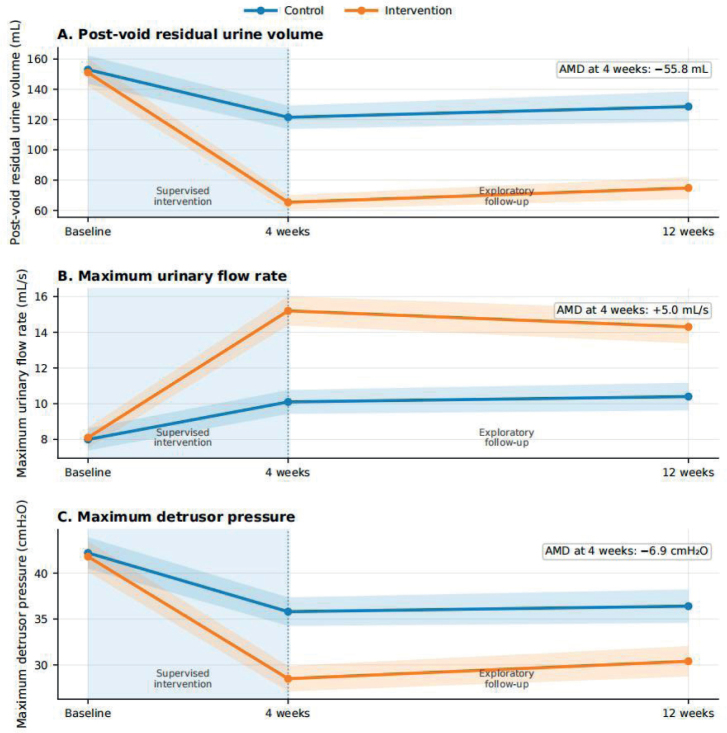
Time course and durability of bladder-function outcomes. Model-style summary plot showing changes in key urodynamic outcomes from baseline to the 4-week follow-up and exploratory 12-week follow-up. (A) Post-void residual urine volume, (B) maximum urinary flow rate, and (C) maximum detrusor pressure. Points represent group means, and shaded bands indicate 95% confidence intervals. The shaded background from baseline to 4 weeks denotes the supervised intervention period; the period from 4 to 12 weeks denotes exploratory post-intervention follow-up. Lower values indicate improvement for post-void residual urine volume and maximum detrusor pressure, whereas higher values indicate improvement for maximum urinary flow rate. Adjusted mean differences at 4 weeks were estimated using ANCOVA models adjusted for baseline values.

At the 4-week follow-up, the intervention group showed greater short-term improvement in the primary outcome than the control group. Mean RUV decreased from 151.1 ± 42.5 mL at baseline to 65.3 ± 22.1 mL at 4 weeks in the intervention group and from 153.0 ± 44.2 mL to 121.5 ± 35.8 mL in the control group. The adjusted mean difference in RUV change was –55.8 mL (95% CI, –64.7 to –46.9; *p* < 0.001). Maximum urinary flow rate and maximum detrusor pressure also improved more in the intervention group than in the control group after multiplicity adjustment (Table II; Fig. 2).

**Table II:** Primary and secondary outcomes and adverse events

Outcome	Autonomous bladder training	Routine care control	Effect estimate	*p*-value/interpretation
4-week core outcomes				
RUV change from baseline, mL	–85.8 ± 39.2	–31.5 ± 39.5	AMD –55.8 (95% CI –64.7 to –46.9)	< 0.001
Maximum urinary flow rate change, mL/s	+7.1 ± 3.4	+2.1 ± 3.2	AMD 5.0 (95% CI 4.0 to 6.0)	< 0.001
Maximum detrusor pressure change, cmH_2_O	–13.3 ± 6.8	–6.4 ± 7.1	AMD –6.9 (95% CI –8.9 to –4.9)	< 0.001
NBSS change from baseline	–9.6 ± 5.6	–3.0 ± 5.2	AMD –6.5 (95% CI –8.1 to –4.9)	< 0.001
Symptomatic UTI requiring antibiotics	8/84 (9.5%)	23/84 (27.4%)	RR 0.35 (95% CI 0.17 to 0.74)	0.003
4-week clinically meaningful outcomes				
RUV reduction >= 50 mL	67/84 (79.8%)	36/84 (42.9%)	RR 1.86 (95% CI 1.42 to 2.43)	< 0.001
RUV < 100 mL at 4 weeks	75/84 (89.3%)	26/84 (31.0%)	RR 2.88 (95% CI 2.08 to 3.99)	< 0.001
NBSS reduction >= 5 points	61/84 (72.6%)	34/84 (40.5%)	RR 1.79 (95% CI 1.34 to 2.39)	< 0.001
Catheterization frequency reduced by >= 1/day	46/84 (54.8%)	24/84 (28.6%)	RR 1.92 (95% CI 1.30 to 2.83)	0.001
Exploratory 12-week outcomes				
RUV at 12 weeks, mL	74.8 ± 31.5	128.6 ± 42.7	AMD –53.2 mL	Partial maintenance
NBSS at 12 weeks	30.1 ± 7.0	36.2 ± 7.8	AMD –6.0	Lower symptom burden
Recurrent symptomatic UTI requiring antibiotics	9/80 (11.3%)	18/79 (22.8%)	RR 0.49	Interpret cautiously

Values are mean ± SD or *n*/*N* (%) unless otherwise indicated. Change was calculated as follow-up value minus baseline value. Negative values indicate improvement for RUV, maximum detrusor pressure, and NBSS; positive values indicate improvement for maximum urinary flow rate. Four-week continuous outcomes are adjusted mean differences from ANCOVA models adjusted for baseline values. Responder thresholds and 12-week outcomes were used to support clinical interpretation and were not adjusted for multiplicity. Culture confirmation was incomplete for symptomatic UTI episodes; infection-related outcomes should be interpreted cautiously.

AMD: adjusted mean difference; CI: confidence interval; NBSS: Neurogenic Bladder Symptom Score; RR: risk ratio; RUV: residual urine volume; UTI: urinary tract infection.

### Secondary and clinically meaningful outcomes

During the 4-week follow-up, clinically diagnosed symptomatic UTI requiring antibiotic treatment occurred in 8 of 84 participants (9.5%) in the intervention group and 23 of 84 participants (27.4%) in the control group (risk ratio, 0.35; 95% CI, 0.17 to 0.74; *p* = 0.003). Urine culture was obtained in 5 of 8 symptomatic episodes (62.5%) in the intervention group and 14 of 23 symptomatic episodes (60.9%) in the control group. Culture-positive symptomatic UTI was observed in 4 participants (4.8%) in the intervention group and 11 participants (13.1%) in the control group. Because urine culture was not systematically available for all episodes, culture-confirmed UTI was considered a supportive exploratory outcome. Episodes of pyuria or bacteriuria without clinical symptoms were not considered symptomatic UTI.

**Fig. 3:**
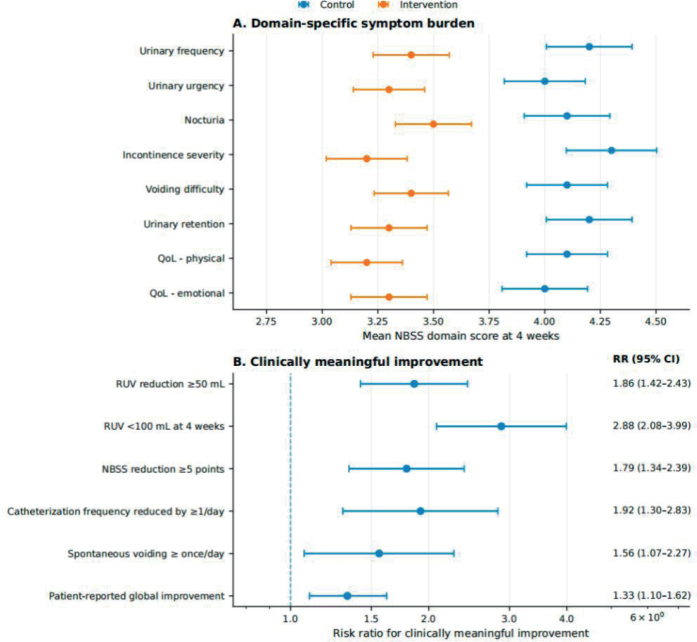
Patient-reported and clinically meaningful improvements at 4 weeks. (A) Domain-specific Neurogenic Bladder Symptom Score (NBSS) scores at the 4-week follow-up. Points represent group means, and horizontal lines indicate 95% confidence intervals. Lower scores indicate fewer symptoms and better disease-specific quality of life. (B) Responder analysis of clinically meaningful outcomes at 4 weeks. Points represent risk ratios, and horizontal lines indicate 95% confidence intervals. The vertical dashed line indicates no between-group difference; values greater than 1 favour autonomous bladder training. Responder outcomes included residual urine volume (RUV) reduction ≥ 50 mL, RUV < 100 mL at 4 weeks, NBSS reduction ≥ 5 points, catheterization frequency reduced by ≥ 1/day, spontaneous voiding ≥ once/day, and patient-reported global improvement.

At 4 weeks, RUV reduction of at least 50 mL was observed in 67 participants (79.8%) in the intervention group and 36 participants (42.9%) in the control group. RUV below 100 mL was achieved by 75 participants (89.3%) in the intervention group compared with 26 participants (31.0%) in the control group. NBSS reduction of at least 5 points occurred in 61 participants (72.6%) in the interFigvention group and 34 participants (40.5%) in the control group. Catheterization frequency was reduced by at least once per day in 46 participants (54.8%) and 24 participants (28.6%), respectively, and spontaneous voiding at least once per day was reported by 42 participants (50.0%) and 27 participants (32.1%), respectively (Table II and Fig. 3).

### Intervention adherence, feasibility, and safety

Seventy-two of 84 participants (85.7%) completed at least 80% of prescribed sessions, and the median overall adherence was 88% (IQR, 82% to 94%). Three participants (3.6%) required temporary interruption of one or more sessions because of transient discomfort or autonomic dysreflexia, but no participant permanently discontinued the intervention. The estimated additional time required was 28 ± 9 min per day for therapist-delivered components and 12 ± 5 min per day for nursing support.

Two participants (2.4%) in the intervention group experienced transient autonomic dysreflexia during desensitization training, and 1 participant (1.2%) had self-limited haematuria. No serious adverse events occurred. Adverse events were collected systematically using predefined monitoring forms in both groups.

### Exploratory analyses

**Fig. 4:**
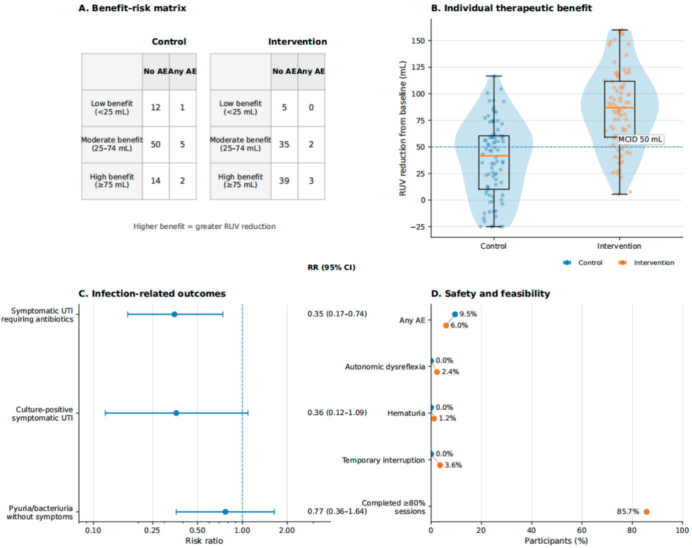
Integrated benefit–risk profile of autonomous bladder training. (A) Benefit–risk matrix categorizing participants according to the magnitude of residual urine volume (RUV) reduction and adverse-event status at 4 weeks. Higher benefit categories indicate greater RUV reduction. (B) Distribution of individual RUV reduction from baseline to 4 weeks. The dashed horizontal line indicates the clinically meaningful threshold of 50 mL. (C) Infection-related outcomes, distinguishing clinically diagnosed symptomatic urinary tract infection (UTI) requiring antibiotic treatment, culture-positive symptomatic UTI, and pyuria/bacteriuria without symptoms. Points represent risk ratios, and horizontal lines indicate 95% confidence intervals; values less than 1 favour autonomous bladder training. (D) Safety and feasibility outcomes, including any adverse event, autonomic dysreflexia, haematuria, temporary interruption, and completion of at least 80% of prescribed sessions. Infection-related outcomes should be interpreted cautiously because urine culture confirmation was incomplete.

Among participants with SCI, the between-group difference in RUV change was –58.3 mL, whereas among participants with non-SCI aetiologies the corresponding difference was –51.3 mL. There was no evidence of a treatment-by-aetiology interaction for the primary outcome (interaction *p* = 0.62). Because of the limited number of non-SCI participants, these subgroup findings should be interpreted as exploratory. Exploratory adherence analysis suggested that participants with adherence of at least 80% tended to have greater RUV reduction than those with lower adherence. Because this analysis was not powered for adherence-response inference and was limited to the intervention group, it was interpreted descriptively rather than as confirmatory evidence of a linear dose–response relationship (Fig. 3). The integrated benefit–risk profile, including individual RUV reduction, infection-related outcomes, and safety and feasibility measures, is summarized in Fig. 4.

Follow-up at 12 weeks was completed in 80 of 84 participants (95.2%) in the intervention group and 79 of 84 participants (94.0%) in the control group. RUV assessment at 12 weeks was available in 72 participants in the intervention group and 70 participants in the control group. The intervention group maintained a lower mean RUV than the control group at 12 weeks (74.8 ± 31.5 mL vs 128.6 ± 42.7 mL), although RUV was slightly higher than at the 4-week assessment. NBSS remained lower in the intervention group (30.1 ± 7.0 vs 36.2 ± 7.8), and daily catheterization frequency was also lower (3.1 ± 1.0 vs 4.0 ± 1.2). Recurrent clinically diagnosed symptomatic UTI requiring antibiotic treatment occurred in 9 of 80 participants (11.3%) in the intervention group and 18 of 79 participants (22.8%) in the control group. UTI-related hospitalization was uncommon in both groups (see Table II).

## DISCUSSION

This randomized controlled trial showed that a structured, multimodal programme of autonomous bladder training, when integrated into routine NB rehabilitation care, improved short-term urodynamic parameters and patient-reported symptoms over 4 weeks. The primary outcome, change in RUV, favoured the intervention group, and exploratory 12-week follow-up suggested partial maintenance of benefit after completion of the supervised intervention period.

The improvement in RUV may reflect the combined effects of complementary training components. Induced voiding and sensory awareness training may enhance recognition of bladder filling and voluntary initiation of voiding. Pelvic floor muscle training may improve voluntary sphincter control, whereas bladder–sphincter coordination exercises may facilitate more coordinated relaxation during voiding. Bladder desensitization with controlled saline instillation may also influence bladder sensation and tolerance (1, 14–16). However, because these components were delivered together as a single integrated programme, the present trial cannot determine the independent contribution of each component.

The lower rate of clinically diagnosed symptomatic UTI requiring antibiotic treatment is clinically relevant because recurrent infection is a major contributor to morbidity and antibiotic exposure in patients with NB (17, 18). This finding may be partly explained by improved bladder emptying and reduced urinary stasis. However, it should be interpreted cautiously. In patients with NB, particularly those managed with CIC, pyuria and bacteriuria are common and do not necessarily indicate symptomatic infection. In addition, urine culture was not systematically available for all clinically diagnosed episodes, and the intervention included saline bladder instillation, which may have influenced leukocyte or bacterial counts through a mechanical washout effect. Therefore, the infection-related findings should be regarded as supportive and exploratory rather than definitive evidence of reduced culture-confirmed UTI.

The exploratory 12-week follow-up provides preliminary evidence that part of the short-term benefit may persist beyond the supervised 4-week intervention period. The intervention group maintained lower RUV and NBSS values and reported lower catheterization frequency than the control group. However, RUV appeared slightly higher at 12 weeks than at the end of the 4-week intervention, suggesting that the effect may be attenuated when supervised training is reduced. This finding supports the need to evaluate maintenance or home-based training strategies.

The intervention demonstrated a favourable safety and tolerability profile. Adverse events were infrequent, mild, and transient, with no serious complications related to the training reported. This supports the feasibility and acceptability of integrating such a structured programme into routine clinical practice. High patient satisfaction rates further suggest that the training was well received and perceived as beneficial by participants. (Qualitative analysis of patient feedback corroborates this, with keywords such as “control”, “confidence”, and “improvement” being most frequently reported, as detailed in Table SIII).

### Limitations

Several limitations should be acknowledged. First, the single-centre design and specialized rehabilitation setting may limit generalizability. Second, the multimodal nature of the intervention limits interpretation of the independent contribution of each component. The trial evaluated the effectiveness of the integrated programme rather than the efficacy of any individual component; the relative contribution, minimum effective dose, and necessity of each component remain uncertain. Third, urine culture confirmation was incomplete for symptomatic UTI episodes, and the timing of urine sampling relative to catheterization and saline instillation could not be fully standardized in all cases. Fourth, although exploratory 12-week follow-up was added, this duration remains insufficient to determine durable bladder function recovery, recurrent UTI prevention, renal function preservation, upper urinary tract outcomes, or long-term catheterization independence. Fifth, the study population was dominated by SCI-related NB. Exploratory subgroup analyses suggested a consistent direction of benefit in SCI and non-SCI participants, but the study was not powered to detect differential treatment effects by aetiology.

Future research should validate these findings in multicentre trials with longer follow-up, ideally 6 to 12 months, and with standardized symptom- and culture-based UTI definitions, prespecified colony-count thresholds, renal function assessment, upper urinary tract imaging, and catheterization outcomes. Factorial trials, dismantling designs, or stepped implementation studies are needed to identify the most active components of the intervention and determine whether simplified or home-based maintenance programmes can sustain benefit with lower resource requirements.

### Conclusion

In this single-centre randomized trial, autonomous bladder training improved short-term urodynamic parameters and patient-reported symptoms over 4 weeks in patients with NB. Exploratory 12-week follow-up suggested partial maintenance of benefit, although some attenuation of RUV improvement was observed after completion of supervised training. The lower rate of clinically diagnosed symptomatic UTI requiring antibiotic treatment is encouraging but should be interpreted cautiously because culture confirmation was incomplete. These findings support further evaluation of this multimodal programme in longer, multicentre trials before broad implementation into routine NB rehabilitation.

## Supplementary Material






