# Supplementary material for: AUTONOMOUS BLADDER TRAINING FOR NEUROGENIC BLADDER: A RANDOMIZED CONTROLLED TRIAL
Source: J Rehabil Med. 2026 Jul 23;58:45818. doi: 10.2340/jrm.v58.45818 (PMC13403163; doi:10.2340/jrm.v58.45818)
Supplement: Supplementary file 1 [file JRM-58-45818-s1.pdf]

Appendix S1

Supplementary Methods
Protocol-specified outcomes and statistical analysis plan summary
The prespecified primary outcome was the change in post-void residual urine volume (RUV) from baseline to the 4-week follow-up. Key secondary urodynamic outcomes were changes in maximum urinary flow rate and maximum detrusor pressure. Other secondary outcomes included clinically diagnosed symptomatic urinary tract infection (UTI) requiring antibiotic treatment, Neurogenic Bladder Symptom Score (NBSS), patient satisfaction, and adverse events. Exploratory outcomes included responder outcomes, 12-week post-intervention outcomes, and subgroup analyses by neurological etiology.

Clinically diagnosed symptomatic UTI requiring antibiotic treatment was defined as new or worsening urinary tract-related symptoms or systemic symptoms judged by the treating physician to be compatible with UTI, together with supportive urinalysis findings and initiation of antibiotic therapy. Urine culture was obtained when clinically indicated; however, culture confirmation was not systematically available for all suspected episodes. Therefore, culture-positive symptomatic UTI was reported as a supportive exploratory outcome rather than as the primary infection definition.

The primary analysis followed the intention-to-treat principle. Continuous outcomes were analyzed using analysis of covariance with treatment group as the main effect and the corresponding baseline value as a covariate. The primary outcome was tested at a two-sided alpha level of 0.05. Holm-Bonferroni adjustment was applied to key secondary urodynamic outcomes. Other secondary and exploratory outcomes, including responder outcomes, symptomatic UTI, 12-week outcomes, and patient-reported outcomes, were interpreted cautiously and exploratorily.

Participant retention and outcome-level data completeness were assessed separately. For continuous outcomes with missing 4-week values, multiple imputation by chained equations was performed under a missing-at-random assumption. The imputation model included treatment group, baseline value of the outcome, age, sex, etiology of neurogenic bladder, disease phase, baseline RUV, catheterization method, and other available 4-week outcomes. Twenty imputed datasets were generated and pooled using Rubin's rules. Complete-case analyses and a conservative worst-case analysis for the primary outcome were performed as sensitivity analyses. Exploratory 12-week outcomes were analyzed descriptively to assess durability of benefit. These outcomes included RUV, NBSS, catheterization frequency, recurrent clinically diagnosed symptomatic UTI requiring antibiotic treatment, UTI-related hospitalization, and late adverse events. The 12-week analyses were not adjusted for multiplicity and were not considered confirmatory.

Safety outcomes were collected systematically using predefined monitoring forms in both groups. Prespecified adverse events included symptomatic UTI requiring antibiotic treatment, autonomic dysreflexia, hematuria, catheter-related trauma, suprapubic pain, hemodynamic instability, and events requiring interruption or discontinuation of the intervention. Serious adverse events were defined as events resulting in death, life-threatening deterioration, prolonged hospitalization, persistent disability, or other medically important conditions
