# Supplementary material for: AUTONOMOUS BLADDER TRAINING FOR NEUROGENIC BLADDER: A RANDOMIZED CONTROLLED TRIAL
Source: J Rehabil Med. 2026 Jul 23;58:45818. doi: 10.2340/jrm.v58.45818 (PMC13403163; doi:10.2340/jrm.v58.45818)

ig. S1. **Domain-Specific Comparison of Neurogenic Bladder Symptom Scores After Intervention.** Parallel coordinates
ymptom Score (NBSS) for each of the 8 domains between the control (blue, n=84) and intervention (orange, n=84) groups
ıean scores across all domains for one group. Error bars represent ± 1 standard deviation around each domain mean. The consi
ne across all domains indicates a comprehensive reduction in symptom burden following the autonomous bladder training prog

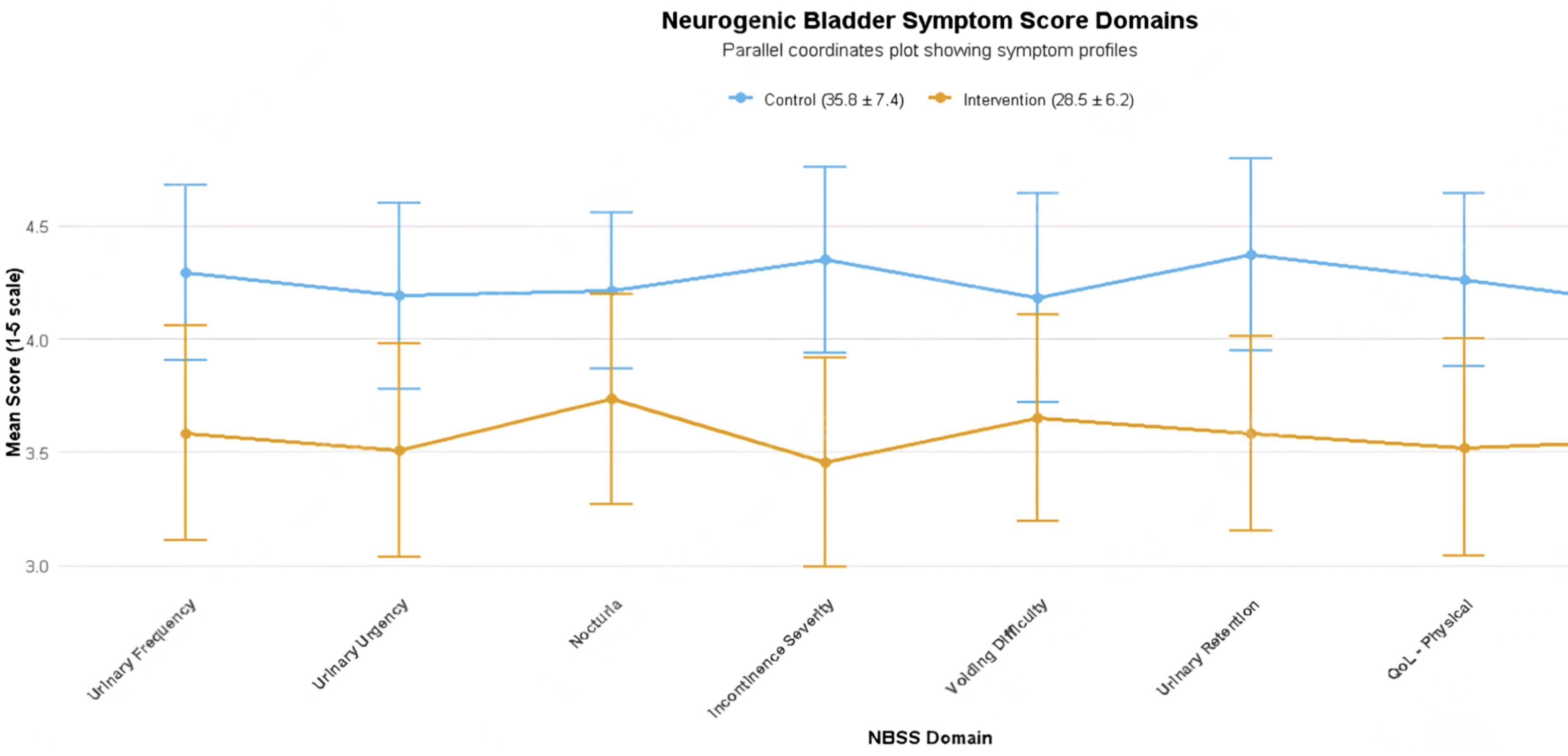

ig. S2. **Comprehensive Benefit-Risk Profile of Autonomous Bladder Training.** Multi-panel exploratory figure comparing ladder training and routine care control groups. Panel A shows specific adverse event types. Panel B categorizes part dverse-event status. Panel C shows individual participant data relating RUV reduction to adverse-event occurrence; dashed ver UV reduction values were aligned with the revised manuscript (85.8 ± 39.2 mL vs. 31.5 ± 39.5 mL). Infection-related finding ulture confirmation was incomplete.

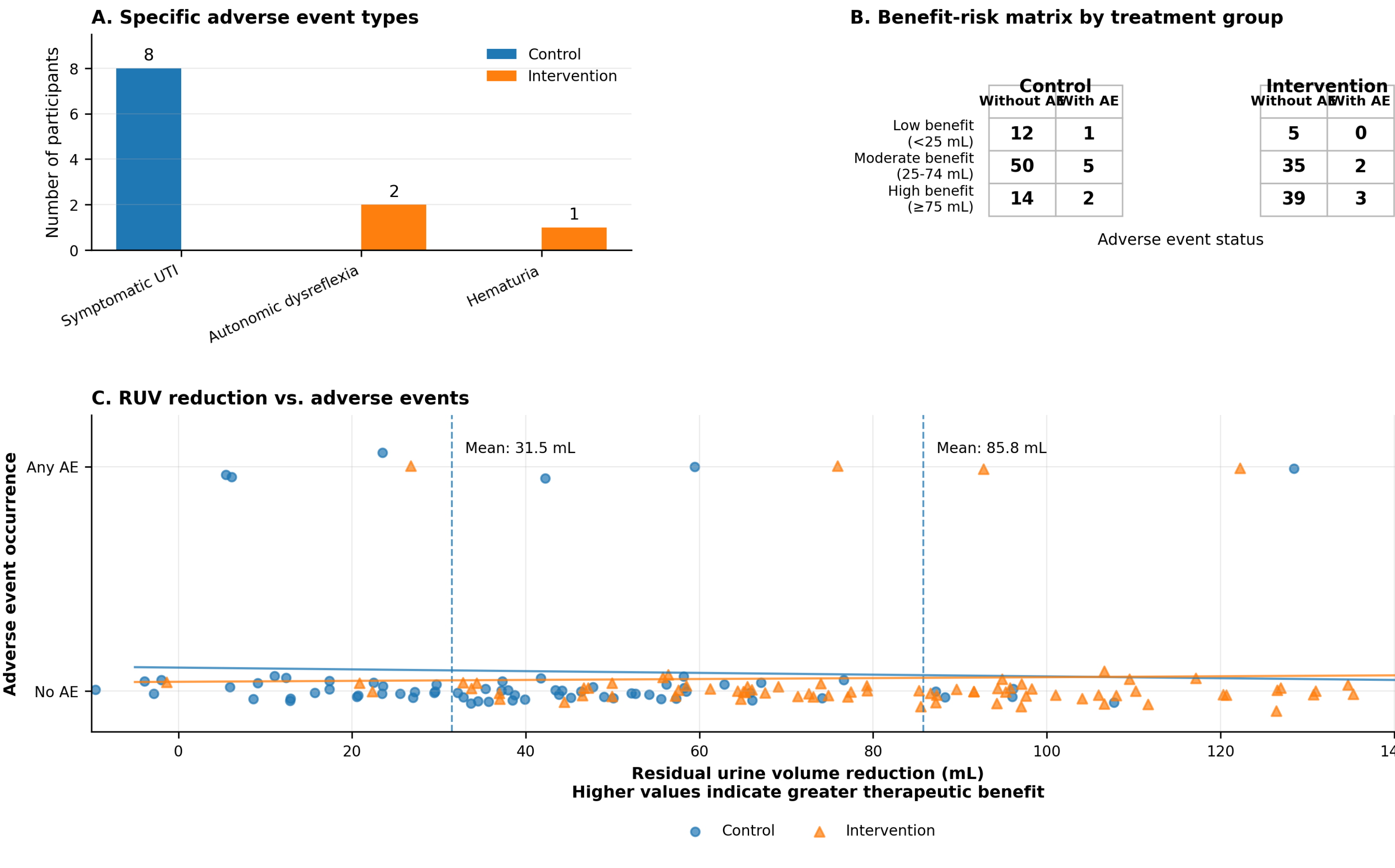

ig. S3. Exploratory adherence and RUV reduction. Scatter plot showing the exploratory association between training adh

olume (RUV) within the autonomous bladder training group. The solid line and shaded area provide a visual linear fit and

mited to the intervention group, and not powered to establish a confirmatory dose-response relationship. The dashed horizon

ɛduction threshold of 50 mL.

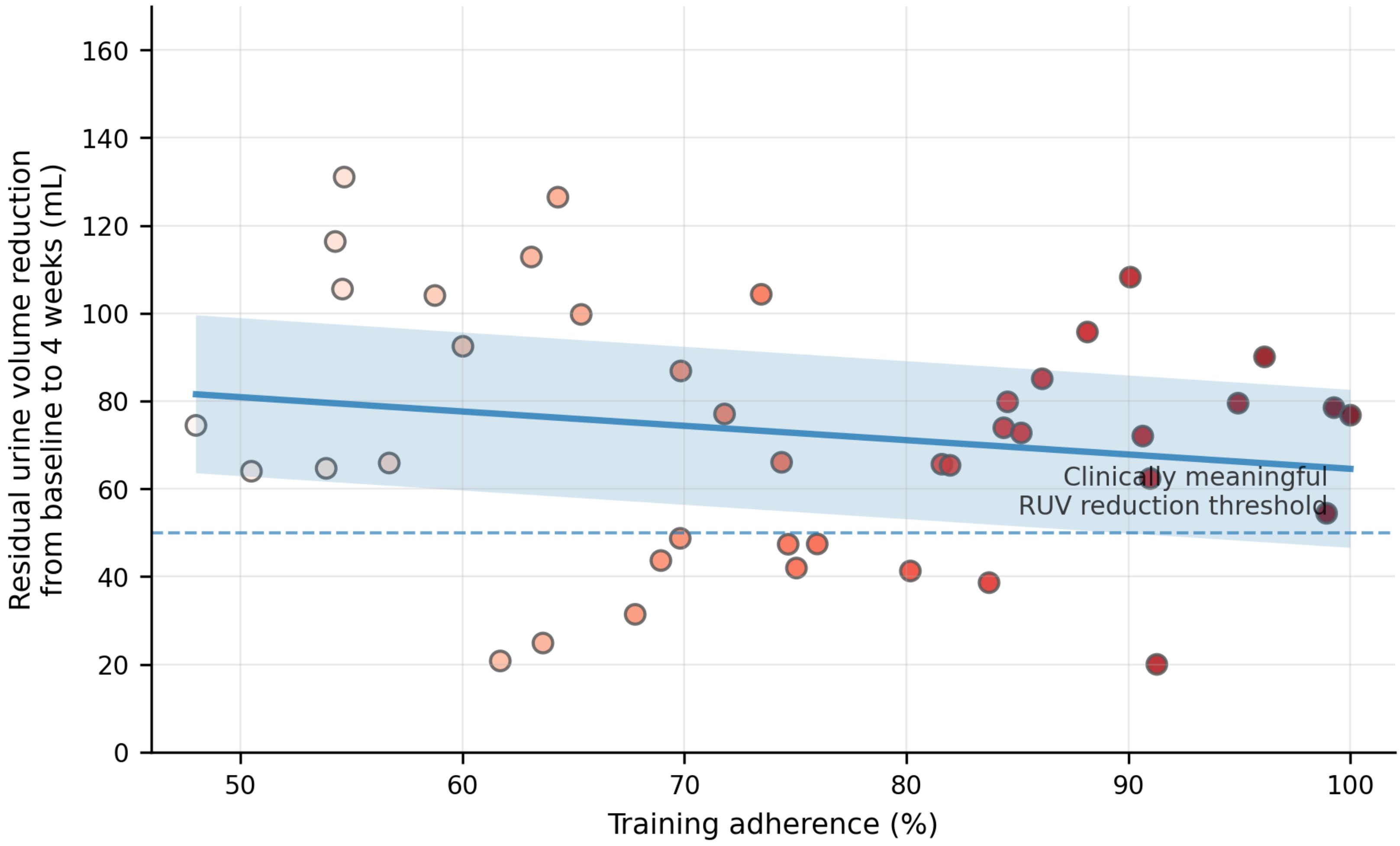

Supplement: Supplementary file 2 [file JRM-58-45818-s2.pdf]
