# Supplementary material for: AUTONOMOUS BLADDER TRAINING FOR NEUROGENIC BLADDER: A RANDOMIZED CONTROLLED TRIAL
Source: J Rehabil Med. 2026 Jul 23;58:45818. doi: 10.2340/jrm.v58.45818 (PMC13403163; doi:10.2340/jrm.v58.45818)
Supplement: Supplementary file 3 [file JRM-58-45818-s3.pdf]

'able SI. Eligibility Criteria for the Randomized Controlled Trial

Category	Criteria	Operational Definition / Notes
Inclusion Criteria	1. Diagnosis	Adult patients (age ≥18 years) diagnosed with neurogenic bladder according to the Comp Lower Urinary Tract Dysfunction in Patients with Neurogenic Bladder.
	2. Disease Phase	Presenting during the subacute (1-6 months post-neurological injury) or chronic phase (>
	3. Clinical Manifestations	At least one of the following: · Post-void residual urine volume (RUV) >100 mL · Uri
	4. Hemodynamic Stability	Absence of sustained hypotension (systolic BP <90 mmHg) or uncontrolled hypertension
	5. Informed Consent	Written informed consent provided by the patient or their legally authorized representativ
Exclusion Criteria	1. Severe Comorbidities	Significant acid-base or electrolyte imbalances, or impaired function of major organs (he
	2. Active Infections & Disorders	Active severe urinary tract infection (UTI), coagulation disorders, or communicable dise
	3. Prior Urological Surgery	History of major urological surgery (e.g., urethral sphincterotomy, cystostomy, bladder a
	4. Bladder Anatomical Issues	Bladder safe capacity <100 mL or presence of bladder perforation.
	5. High-Risk Urodynamic Factors	Frequent autonomic dysreflexia (AD), sustained bladder pressure >40 cmH_2O, or vesico
	6. Cognitive/Psychiatric Instability	Impaired consciousness (e.g., coma), diagnosed psychiatric disorders, or clinically unsta
	7. Fluid Management	Requirement for large-volume fluid resuscitation during the study period.
	8. Non-compliance	Unwillingness to provide informed consent or comply with study procedures and follow-
	9. Concurrent Participation	Participation in another interventional clinical trial within the past 30 days.
	10. Life Expectancy	Life expectancy <6 months due to any comorbidity (e.g., metastatic cancer, end-stage or

[illegible]able SII. Detailed Protocol of the Multimodal Autonomous Bladder Function Training Program

Training Component	Specific Procedure	Frequency	Duration per Se[illegible]
Induced Voiding	Auditory stimulation (playing running water sounds) combined with thermal stimulation (application of a warm pack) to the suprapubic region to trigger the micturition reflex.	Before each scheduled catheterization	5–10 minutes
Voiding Awareness (Sensory Training)	Guided mindfulness and imagery exercise focused on bladder sensation and voluntary voiding, performed while seated in a voiding position.	Prior to each catheterization	5 minutes
Pelvic Floor Muscle Training	Sustained contraction of the anal sphincter (as a proxy for pelvic floor muscles) held for 5–10 seconds, followed by complete relaxation. One set consists of 10–20 repetitions.	3 sets per day	10–20 minutes [illegible] (including rest i[illegible]
Bladder-Sphincter Coordination Training	Digital anal stretching performed by a trained therapist in four directional quadrants (clockwise, counterclockwise, anterior, posterior) to reduce sphincter spasticity and improve synergy with detrusor contraction.	5 sessions per day	10 stretches per [illegible] (~2–3 minutes) [illegible]
Bladder Desensitization Training	Post-void instillation of 100 mL of sterile saline into the bladder via catheter, alternating between warmed (37–40°C) and room-temperature (20–25°C) saline daily. The fluid is retained for 5 minutes before drainage.	Once daily	15–20 minutes (including instil[illegible] retention, and d[illegible]

[illegible]his structured program was delivered daily under the supervision of trained rehabilitation staff over a 4-week period. All comp[illegible]
[illegible]are plan and documented in a standardized therapy log.

'able SIII. Patient-Reported Keywords from Open-Ended Feedback in the Autonomous Bladder Training Group

Rank	Keyword (English)	Keyword (Chinese)	Frequency (n=84)	Representative Quote (Translated)
1	Control	控制感	68	I feel more in control of my bladder now.
2	Confidence	自信	62	This training gave me back my confidence.
3	Improvement	改善	58	I've noticed a real improvement in my sympto
4	Independence	独立	55	I rely less on others for catheterization.
5	Routine	规律	48	The daily routine gave me structure and hope.
6	Awareness	意识	45	I'm more aware of when I need to go.
7	Hope	希望	42	For the first time, I feel hopeful about the futu
8	Effort	费力	38	The exercises require a lot of effort every day.
9	Time-consuming	耗时	35	It's time-consuming, but worth it.
10	Discomfort	不适	28	Some of the stretching caused temporary disco
11	Confusing	困惑	18	The instructions were confusing at first.
12	Burden	负担	15	Adding this to my daily care felt like a burden
13	Frustration	沮丧	12	There were days of frustration when progress
14	Pain	疼痛	8	I experienced some pain during the desensitiza
15	Uncertainty	不确定	6	I'm still uncertain if this will work long-term.

'ord Cloud of Patient-Reported Experiences Following Autonomous Bladder Training. Word cloud generated from open-endec .utonomous Bladder Training group (n=84). The size of each word corresponds to its frequency of appearance in the qualitative Confidence," "Improvement") represent the most commonly reported themes. The visualization highlights that positive themes nprovement dominated the patient experience, providing qualitative context for the quantitative improvements in clinical outco

able SIV. Completeness of outcome data and missingness pattern

Outcome	Autonomous bladder training available/missing	Routine care contr[illegible]
RUV at 4 weeks	Jan-83	Jan[illegible]
Maximum urinary flow rate at 4 weeks	Jan-83	Feb[illegible]
Maximum detrusor pressure at 4 weeks	Feb-82	Feb[illegible]
NBSS total score at 4 weeks	Jan-83	Jan[illegible]
Patient satisfaction at 4 weeks	84/0	Jan[illegible]
Clinically diagnosed symptomatic UTI	84/0	84[illegible]
Adverse-event status	84/0	84[illegible]
Completed 12-week follow-up	Apr-80	May[illegible]
RUV assessment at 12 weeks	Dec-72	70/[illegible]

rine culture confirmation among symptomatic UTI episodes was not treated as missing outcome data because culture was not s[illegible] iagnosed episodes and was reported separately as supportive exploratory evidence.
